# Complete Genome Sequence of Burkholderia cenocepacia Phage Magia

**DOI:** 10.1128/MRA.01473-20

**Published:** 2021-02-11

**Authors:** David Gafford-Gaby, Guichun Yao, Tram Le, James Clark, Carlos Gonzalez, Jason Gill, Mei Liu

**Affiliations:** a Center for Phage Technology, Texas A&M University, College Station, Texas, USA; Queens College

## Abstract

Burkholderia cenocepacia is a Gram-negative bacterium that is implicated in respiratory infections. The 44,942-bp genome of Magia, a phage infecting B. cenocepacia, does not appear to have strong overall similarity to other known phages. The Magia genome encodes a Cro-like transcriptional regulator, a C2-like immunity repressor, and an integrase, suggesting that it is a temperate phage.

## ANNOUNCEMENT

Burkholderia cenocepacia is a Gram-negative bacterium that typically resides in soil (1, 2). It is an opportunistic pathogen that causes aggressive respiratory infections in individuals with cystic fibrosis and chronic granulomatous disease (1, 3, 4). Study of phage Magia may help advance phage clinical applications for the treatment of *Burkholderia* infections.

Phage Magia was isolated from Nebraska Sandhills soil samples collected from Holt County, Nebraska, in 2018 and was propagated on B. cenocepacia strain MS1 grown aerobically in tryptic soy broth/agar at 37°C. Phage isolation and propagation were performed using the soft-agar overlay method (5). DNA was purified following a previously described protocol using the Promega Wizard DNA kit (6), prepared for sequencing with 550-bp inserts using a TruSeq Nano kit (Illumina), and sequenced as paired-end 250-bp reads by Illumina MiSeq sequencing with 500-cycle v2 chemistry. Sequencing raw reads (1,182,570 reads in total) obtained from the library index were checked for quality using FastQC (www.bioinformatics.babraham.ac.uk/projects/fastqc), and the genome was assembled, using SPAdes v3.5.0 (7), from these reads to a contig corresponding to phage Magia with 21-fold coverage. The end sequences of the assembled genome were verified by PCR using PCR primers (5′-ATCATCACCTCTTCGCACTG-3′ and 5′-GTAACGGAGAACAGTACGAGC-3′) facing off the contig ends and Sanger sequencing of the resulting PCR product. Genes were identified using GLIMMER v3 (8) and MetaGeneAnnotator v1.0 (9), and tRNAs were identified using ARAGORN v2.36 (10). The identified genes were assigned putative functions using BLAST v2.9.0 with the nonredundant and Swiss-Prot databases (11, 12), InterProScan v5.33 (13), and TMHMM v2.0 (14). For comparative purposes, whole-genome DNA sequence similarity was assessed using progressiveMauve v2.4 (15). All analyses were conducted using tools hosted on the Center for Phage Technology (CPT) Galaxy and Apollo interfaces (16–18) with default settings (https://cpt.tamu.edu/galaxy-pub).

B. cenocepacia phage Magia has a 44,942-bp genome with a GC content of 65.1%, approximately equivalent to that of its host (19). Seventy protein-encoding genes were identified in phage Magia, with a coding density of 95.4%; of these, putative functions were assigned for 33 proteins. Phage Magia does not appear to be closely related to any other reported phages. Its closest match is *Burkholderia* phage Bups phi1 (GenBank accession numbers EU307292.1 to EU307295.1), with 24.91% nucleotide identity and 33 similar unique proteins, as determined by progressiveMauve and BLASTp, with an E value cutoff of 0.001. A programmed translational frameshift in the tape measure chaperone gene was identified, and the presence of baseplate and tail sheath proteins suggests that the phage is a myophage. Notably, the Magia genome encodes a Cro-like transcriptional regulator, a C2-like immunity repressor, and an integrase, suggesting that it is a temperate phage. The Magia genome is also related to elements in *Burkholderia* genomes (e.g., 97% BLASTn identity to the position 3619498 to 3634698 region of the Burkholderia cepacia DDS 7H-2 chromosome [GenBank accession number CP007787]). A potential diversity-generating retroelement (DGR), including reverse transcriptase and Avd proteins, was identified in Magia. These systems can introduce variability into the genome via mutagenic retrohoming (20); potential mutagenesis target sequences and initiation of mutagenic homing (IMH) regions were identified in the relatively short reading frames of genes *47* and *57*. This system may allow the phage to adapt to rapidly changing conditions or to alter its host range (21).

### Data availability.

The genome sequence of phage Magia was deposited under GenBank accession number MT701587 and BioSample accession number SAMN14609636. The BioProject accession number is PRJNA222858, and the SRA accession number is SRR11558336.

## References

[1] Brackman G, Hillaert U, Van Calenbergh S, Nelis HJ, Coenye T 2009 Use of quorum sensing inhibitors to interfere with biofilm formation and development in *Burkholderia multivorans* and *Burkholderia cenocepacia*. Res Microbiol 160:144–151. doi:10.1016/j.resmic.2008.12.003. 19146953

[2] Coenye T, Vandamme P 2003 Diversity and significance of *Burkholderia* species occupying diverse ecological niches. Environ Microbiol 5:719–729. doi:10.1046/j.1462-2920.2003.00471.x. 12919407

[3] Drevinek P, Mahenthiralingam E 2010 *Burkholderia cenocepacia* in cystic fibrosis: epidemiology and molecular mechanisms of virulence. Clin Microbiol Infect 16:821–830. doi:10.1111/j.1469-0691.2010.03237.x. 20880411

[4] Baldwin A, Mahenthiralingam E, Drevinek P, Vandamme P, Govan JR, Waine DJ, LiPuma JJ, Chiarini L, Dalmastri C, Henry DA, Speert DP, Honeybourne D, Maiden MCJ, Dowson CG 2007 Environmental *Burkholderia cepacia* complex isolates from human infections. Emerg Infect Dis 13:458–461. doi:10.3201/eid1303.060403. 17552100 PMC2725883

[5] Chen M, Zhang L, Abdelgader SA, Yu L, Xu J, Yao H, Lu C, Zhang W 2017 Alterations in gp37 expand the host range of a T4-like phage. Appl Environ Microbiol 83:e01576-17. doi:10.1128/AEM.01576-17. 28939606 PMC5691408

[6] Summer EJ 2009 Preparation of a phage DNA fragment library for whole genome shotgun sequencing. Methods Mol Biol 502:27–46. doi:10.1007/978-1-60327-565-1_4. 19082550

[7] Bankevich A, Nurk S, Antipov D, Gurevich AA, Dvorkin M, Kulikov AS, Lesin VM, Nikolenko SI, Pham S, Prjibelski AD, Pyshkin AV, Sirotkin AV, Vyahhi N, Tesler G, Alekseyev MA, Pevzner PA 2012 SPAdes: a new genome assembly algorithm and its applications to single-cell sequencing. J Comput Biol 19:455–477. doi:10.1089/cmb.2012.0021. 22506599 PMC3342519

[8] Delcher AL, Harmon D, Kasif S, White O, Salzberg SL 1999 Improved microbial gene identification with GLIMMER. Nucleic Acids Res 27:4636–4641. doi:10.1093/nar/27.23.4636. 10556321 PMC148753

[9] Noguchi H, Taniguchi T, Itoh T 2008 MetaGeneAnnotator: detecting species-specific patterns of ribosomal binding site for precise gene prediction in anonymous prokaryotic and phage genomes. DNA Res 15:387–396. doi:10.1093/dnares/dsn027. 18940874 PMC2608843

[10] Laslett D, Canback B 2004 ARAGORN, a program to detect tRNA genes and tmRNA genes in nucleotide sequences. Nucleic Acids Res 32:11–16. doi:10.1093/nar/gkh152. 14704338 PMC373265

[11] The UniProt Consortium. 2018 UniProt: the universal protein knowledgebase. Nucleic Acids Res 46:2699. doi:10.1093/nar/gky092. 29425356 PMC5861450

[12] Camacho C, Coulouris G, Avagyan V, Ma N, Papadopoulos J, Bealer K, Madden TL 2009 BLAST+: architecture and applications. BMC Bioinformatics 10:421. doi:10.1186/1471-2105-10-421. 20003500 PMC2803857

[13] Jones P, Binns D, Chang HY, Fraser M, Li W, McAnulla C, McWilliam H, Maslen J, Mitchell A, Nuka G, Pesseat S, Quinn AF, Sangrador-Vegas A, Scheremetjew M, Yong SY, Lopez R, Hunter S 2014 InterProScan 5: genome-scale protein function classification. Bioinformatics 30:1236–1240. doi:10.1093/bioinformatics/btu031. 24451626 PMC3998142

[14] Krogh A, Larsson B, von Heijne G, Sonnhammer EL 2001 Predicting transmembrane protein topology with a hidden Markov model: application to complete genomes. J Mol Biol 305:567–580. doi:10.1006/jmbi.2000.4315. 11152613

[15] Darling AE, Mau B, Perna NT 2010 progressiveMauve: multiple genome alignment with gene gain, loss and rearrangement. PLoS One 5:e11147. doi:10.1371/journal.pone.0011147. 20593022 PMC2892488

[16] Ramsey J, Rasche H, Maughmer C, Criscione A, Mijalis E, Liu M, Hu JC, Young R, Gill JJ 2020 Galaxy and Apollo as a biologist-friendly interface for high-quality cooperative phage genome annotation. PLoS Comput Biol 16:e1008214. doi:10.1371/journal.pcbi.1008214. 33137082 PMC7660901

[17] Dunn NA, Unni DR, Diesh C, Munoz-Torres M, Harris NL, Yao E, Rasche H, Holmes IH, Elsik CG, Lewis SE 2019 Apollo: democratizing genome annotation. PLoS Comput Biol 15:e1006790. doi:10.1371/journal.pcbi.1006790. 30726205 PMC6380598

[18] Jalili V, Afgan E, Gu Q, Clements D, Blankenberg D, Goecks J, Taylor J, Nekrutenko A 2020 The Galaxy platform for accessible, reproducible and collaborative biomedical analyses: 2020 update. Nucleic Acids Res 48:W395–W402. doi:10.1093/nar/gkaa434. 32479607 PMC7319590

[19] Sass AM, Van Acker H, Förstner KU, Van Nieuwerburgh F, Deforce D, Vogel J, Coenye T 2015 Genome-wide transcription start site profiling in biofilm-grown *Burkholderia cenocepacia* J2315. BMC Genomics 16:775. doi:10.1186/s12864-015-1993-3. 26462475 PMC4603805

[20] Wu L, Gingery M, Abebe M, Arambula D, Czornyj E, Handa S, Khan H, Liu M, Pohlschroder M, Shaw KL, Du A, Guo H, Ghosh P, Miller JF, Zimmerly S 2018 Diversity-generating retroelements: natural variation, classification and evolution inferred from a large-scale genomic survey. Nucleic Acids Res 46:11–24. doi:10.1093/nar/gkx1150. 29186518 PMC5758913

[21] Schillinger T, Zingler N 2012 The low incidence of diversity-generating retroelements in sequenced genomes. Mob Genet Elements 2:287–291. doi:10.4161/mge.23244. 23481467 PMC3575424

